# Polypharmacy in primary care: A population-based retrospective cohort study of electronic health records

**DOI:** 10.1371/journal.pone.0308624

**Published:** 2024-09-04

**Authors:** Thomas Woodcock, Derryn Lovett, Gloria Ihenetu, Vesselin Novov, Thomas Beaney, Keivan Armani, Angela Quilley, Azeem Majeed, Paul Aylin

**Affiliations:** 1 Department of Primary Care and Public Health, School of Public Health, Imperial College London, London, United Kingdom; 2 Department of Primary Care and Public Health, NIHR ARC Northwest London, School of Public Health, Imperial College London, London, United Kingdom; 3 Chelsea And Westminster Hospital NHS Foundation Trust, London, United Kingdom; University of Oxford, UNITED KINGDOM OF GREAT BRITAIN AND NORTHERN IRELAND

## Abstract

**Background:**

Polypharmacy, prescription of multiple medications to a patient, is a major challenge for health systems. There have been no peer-reviewed studies of polypharmacy prevalence and medication cost at a population level in England.

**Aims:**

To determine prevalence and medication cost of polypharmacy, by patient characteristics.

Design and setting: Retrospective cohort study of North West London electronic health records

**Method:**

We quantified prevalence and direct cost of polypharmacy (five or more regular medications), stratified by demographics and frailty. We fitted a mixed-effects logistic regression for polypharmacy.

**Results:**

Of 1.7 million adults, 167,665 (9.4%) were on polypharmacy. Age and socio-economic deprivation were associated with polypharmacy (OR 9.24 95% CI 8.99 to 9.50, age 65–74 compared with 18–44; OR 0.68 95% CI 0.65 to 0.71, least deprived compared with most). Polypharmacy prevalence increased with frailty (OR 1.53 95% CI 1.53 to 1.54 per frailty component, for White women). Men had higher odds of polypharmacy than women at average frailty (OR 1.26 95% CI 1.24 to 1.28) and with additional frailty components (OR 1.10 95% CI 1.09 to 1.10). Black people had lower odds of polypharmacy at average frailty (OR 0.82 95% CI 0.79 to 0.85, compared with White), but along with other ethnicities, saw greater odds increases with increasing frailty (OR 1.02 95% CI 1.01 to 1.03). Annual medication cost 8.2 times more for those on polypharmacy compared with not (£370.89 and £45.31).

**Conclusion:**

Demographic characteristics are associated with polypharmacy, after adjusting for frailty. Further research should explore why, to reduce health inequities and optimise cost associated with polypharmacy.

## Introduction

Polypharmacy, where a patient is taking multiple medications, is a growing area of focus in healthcare [1, 2]. The prevalence of polypharmacy has risen since the 1990s and is expected to continue increasing, along with that of multiple long-term conditions (LTCs), as populations age [3–5]. In England, the average number of prescriptions per person-year has increased from 13 (in 2003) to 19 (in 2013) [6]. Whilst for many patients, polypharmacy is appropriate, for some it is problematic and may cause harm [2]. Indeed, polypharmacy is associated with increased rates of adverse drug events including through harmful drug interactions [7, 8], reduced medication adherence [9], and higher drug costs [10].

A patient is widely accepted as being ‘on polypharmacy’ if they are regularly taking five or more prescription medications [11], although definitions vary by disease focus and clinical setting [1, 2, 11]. However, there are other factors beyond a simple medication count that influence patient outcomes. A 2003 WHO study found that between 30% and 50% of medications for LTCs are not taken as intended [12]. The number of individuals with multiple LTCs in the United Kingdom (UK) has increased [13], so the burden of this poor medication adherence on patients and healthcare systems is likely also greater. People with LTCs such as cardiovascular disease and diabetes have a high prevalence of multimorbidity and associated polypharmacy [14]. LTCs contribute to higher healthcare expenditure, with cardiovascular disease medications among the most commonly prescribed. The impact of polypharmacy on healthcare expenditure has not yet been thoroughly evaluated [15].

Population electronic health record (EHR) databases enable research into the prevalence and nature of polypharmacy, free from biases introduced by sampling or survey-based studies. Such databases capture patients’ medical histories through interactions with multiple healthcare settings. An EHR study of polypharmacy in 180,000 adults in Scotland found 21.5% were prescribed four or more repeat medications, and higher prevalence in patients with more clinical conditions [5]. Another study, of 310,000 adults in Scotland, showed not only that the proportion of adults prescribed 5 or more drugs doubled over 15 years, but an increase in the risk of potentially serious drug-drug interactions [16]. A Spanish study, comparing 66,000 individuals in 2005 to 357,000 individuals in 2015 identified increased use of medications including proton pump inhibitors, statins, and psychotropic drugs [17, 18]. Whilst previous studies of polypharmacy prevalence using EHR data have adjusted for age and sex of patients, none have done so for ethnicity.

A national review of overprescribing published by the Department of Health and Social Care in 2021 found 15% of people in England taking five or more unique medications [19]. However, a 2024 systematic review of the prevalence of multimorbidity and polypharmacy among adults in clinical and community settings identified only small, or health survey based, peer-reviewed studies set in England [20]. There have been no peer-reviewed studies examining prevalence of, and groups most affected by, polypharmacy using population-level EHR data in England. A 2020 rapid review found further research is needed on prevalence and consequences of problematic polypharmacy in the UK. The review also only found evidence of costs of potentially inappropriate polypharmacy outside of the UK. Costs of polypharmacy have been examined in relation to medication reviews in small samples and trials but not at population level [21, 22].

The aims of this study were therefore first, to determine the prevalence of polypharmacy in the population of North West London (NWL), and to quantify associations between patient demographics, including ethnicity and frailty, and polypharmacy. Second, to determine direct medication costs associated with polypharmacy.

## Methods

### Study design, data source and participants

In NWL, local health care providers have worked together to produce the Whole Systems Integrated Care (WSIC) dataset, comprising linked electronic health and social care records covering 2.4 million people registered to GP practices in NWL [23]. We conducted a retrospective cohort study using de-identified WSIC data extracted on 1^st^ November 2023, with study period 1^st^ Jan 2022 to 31^st^ Dec 2022. The authors did not have access to information that could identify individual participants during or after data collection. Participants were 18 years or older, and registered with a GP in NWL, at the start of this period. We linked demographic and disease condition data for every adult to their prescription records for the study period.

### Variables

We defined polypharmacy to be five or more distinct regular medications prescribed to a patient during the study period. To count distinct medications, we used SNOMED CT disposition categories [24]. These dispositions correspond with drug functions within the body. For example, statins form a disposition called “3-hydroxy-3-methylglutaryl-coenzyme A reductase inhibitor”. The link from a prescribed medication to a disposition is made via the medication’s active ingredients, the chemical substance(s) it is formed from. This is illustrated for statins in Table 1. Some active ingredients in the SNOMED CT ontology do not have an assigned disposition. A general practitioner and a pharmacist independently reviewed these active ingredients, and agreed on one of two classifications for each. First, ingredients not relevant for polypharmacy (e.g. mineral oil, alginic acid, virus antigens) were excluded. Others relevant for polypharmacy (e.g. metformin, paracetamol, laxatives), were assigned to an existing SNOMED CT disposition if possible, or a newly created disposition if not. A full list of these assignments is provided in S4 Table.

**Table 1:** Disposition medications: Statins. List of medications prescribed to more than 1000 patients during the study period, associated to the Statin disposition in SNOMED CT with their full list of active ingredients and the number of patients who were prescribed that medication regularly within the study period. Full list available in S5 Table.

Disposition	Medication (Defined as ‘Product’ in SNOMED CT)	Ingredient	Patient Count
**Statin**	Atorvastatin 20mg tablets	Atorvastatin	92,237
	Atorvastatin 40mg tablets		52,988
	Atorvastatin 10mg tablets		19,845
	Atorvastatin 80mg tablets		16,644
	Product containing precisely simvastatin 20 milligram/1 each conventional release oral tablet	Simvastatin	14,955
	Product containing precisely simvastatin 40 milligram/1 each conventional release oral tablet		13,640
	Product containing precisely rosuvastatin (as rosuvastatin calcium) 5 milligram/1 each conventional release oral tablet	Rosuvastatin	5,367
	Product containing precisely rosuvastatin (as rosuvastatin calcium) 10 milligram/1 each conventional release oral tablet		5,121
	Product containing precisely rosuvastatin (as rosuvastatin calcium) 20 milligram/1 each conventional release oral tablet		3,968
	Product containing precisely simvastatin 10 milligram/1 each conventional release oral tablet	Simvastatin	2,748
	Product containing precisely pravastatin sodium 20 milligram/1 each conventional release oral tablet	Pravastatin	2,015
	Product containing precisely pravastatin sodium 40 milligram/1 each conventional release oral tablet		1,705
	Product containing precisely pravastatin sodium 10 milligram/1 each conventional release oral tablet		1,395
	Product containing precisely rosuvastatin (as rosuvastatin calcium) 40 milligram/1 each conventional release oral tablet	Rosuvastatin	1,036

Prescribed items were excluded if they did not contain an active ingredient (e.g. casts and crutches). We also excluded non-oral medications (e.g. injections, gels, ointments; full list in S6 Table), and medications with seven or more active ingredients (all multivitamin medications) which would skew counts. A small number of medications were excluded as not relevant for another reason (S6 Table).

A disposition was deemed to be “regular” for a patient if it was prescribed three or more times for that patient during the study period. Where there were multiple prescriptions of the same disposition for the same patient within 10 days, these were only counted once. The number of regular dispositions identified per patient was used to categorise patients as having 0, 1 to 4, or 5 or more regular medications, the latter defined to be polypharmacy.

Drug costs were taken from the NHS Business Services Authority Dictionary of Medicines and Devices [25]. Recorded drug costs were presented as totals and averages per patient over demographic categories.

We extracted demographic variables for each individual: age, gender, ethnicity, a code for the GP practice the individual was registered with, and Index of Multiple Deprivation (IMD) quintile [26]. IMD is a statistic of relative socio-economic deprivation derived for small areas in England, UK. We extracted information on components of the electronic frailty index (eFI), a tool quantifying frailty in terms of deficits an individual has, as documented in the EHR. The eFI was designed and validated with patients aged 65–95 [27] and contains 36 components including Arthritis, Diabetes, Dizziness, Weight loss and anorexia, and Hypertension. Polypharmacy is also a component of the eFI defined as prescription of ≥5 prescribed medications, using chapters 1–15 of the British National Formulary [27], this was excluded from the analysis in favour of the definition described above.

### Statistical analysis

The unit of observation was the patient. We described demographics stratified by number of regular medications. We used a mixed-effects multivariable logistic regression model to determine factors associated with polypharmacy, modelling variation between GP practices through a random intercept. Age, gender, ethnicity, deprivation quintile and number of eFI components were fixed effects. Missing data for ethnicity were treated as a separate level. Regression diagnostics were performed including examination of residuals, a check for multicollinearity and assessment of the Akaike Information Criterion. Results were reported as odds ratios (ORs) with 95% confidence intervals (CIs), and p-values at a statistical significance level of 0.05. We included interaction terms to examine any modification of the effect of eFI by gender or ethnicity. For all pairs of regular medications, we calculated the proportion of patients prescribed both, and plotted the results as a heatmap.

We performed all statistical analyses using R version 3.6.1.

The NHS Health Research Authority West Midlands—Solihull Research Ethics Committee provided written ethical approval covering this study under the Discover Research Platform (REC: 18/WM/0323, IRAS: 252449), waiving the need for informed consent since the study was retrospective and used de-identified data only. Data access for this study was approved by the North West London Data Access Committee (ID-271-2). All methods within this study were carried out in accordance with the guidelines and regulations of the ethics and study approval.

## Results

A total of 1.7 million patients were included, with 2.3 million regular medications prescribed containing at least one active ingredient. Of these patients, 30 had gender recorded as “Other”. We were unable to break this category down any further to comply with information governance; hence, they are not included in the descriptive analysis. We did not include them in the regression analysis, to avoid complete separation of the model. Ethnicity was missing for 67,680 (3.8% of included) patients. Overall, 9.4% (167,665/1,784,876) of patients were on polypharmacy. A larger proportion of females were on polypharmacy 10.3% (87,847/854,039), compared with males 8.6% (79,818/930,807) (Table 2). Polypharmacy increased in prevalence with age, from 0.9% (9,141/987,776) in those aged 18 to 44, to 51.1% (57,242/112,118) in those 75 and over. Prevalence also increased with deprivation, from 9.2% (12,957/140,488) in the least deprived areas to 11.3% (23,793/210,952) in the most deprived. The ethnicity group with the highest polypharmacy prevalence was Asian, 12.0% (58,404/487,382).

**Table 2:** Study population summary. Study population demographics grouped by the number of regular dispositions they were on within the study period, 0 dispositions, 1 to 4 dispositions, 5 or more dispositions, and total count of patients with that demographic trait.

Demographic Category	Patients with 0 Regular Medications (%)	Patients with 1–4 Regular Medications (%)	Patients with > = 5 Regular Medications (%)	Total Patient Count
**All Patients**	1,267,545 (71.0%)	349,666 (19.6%)	167,665 (9.4%)	1,784,876
**Age Group**				
**18–44**	870,871 (88.2%)	107,764 (10.9%)	9,141 (0.9%)	987,776
**45–64**	334,354 (61.8%)	150,253 (27.8%)	56,013 (10.4%)	540,620
**65–74**	43,201 (29.9%)	55,892 (38.7%)	45,269 (31.4%)	144,362
**75+**	19,119 (17.1%)	35,757 (31.9%)	57,242 (51.1%)	112,118
**Ethnicity**				
**White**	568,704 (69.6%)	172,305 (21.1%)	75,895 (9.3%)	816,904
**Asian**	327,250 (67.1%)	101,728 (20.9%)	58,404 (12.0%)	487,382
**Black**	101,938 (67.9%)	32,193 (21.4%)	16,038 (10.7%)	150,169
**Mixed**	40,059 (73.0%)	10,564 (19.3%)	4,215 (7.7%)	54,838
**Other**	164,200 (79.0%)	30,773 (14.8%)	12,930 (6.2%)	207,903
**Missing**	65,394 (96.6%)	2,103 (3.1%)	183 (0.3%)	67,680
**Gender**				
**Female**	563,737 (66.0%)	202,455 (23.7%)	87,847 (10.3%)	854,039
**Male**	703,781 (75.6%)	147,208 (15.8%)	79,818 (8.6%)	930,807
**IMD Quintile**				
**Most Deprived 1**	143,992 (68.3%)	43,167 (20.5%)	23,793 (11.3%)	210,952
**2**	398,818 (71.1%)	107,541 (19.2%)	54,329 (9.7%)	560,688
**3**	374,139 (71.5%)	100,887 (19.3%)	48,424 (9.3%)	523,450
**4**	254,656 (72.9%)	66,480 (19.0%)	28,162 (8.1%)	349,298
**Least Deprived 5**	95,940 (68.3%)	31,591 (22.5%)	12,957 (9.2%)	140,488

Adjusted effects of age and IMD showed similar patterns to unadjusted prevalence (Table 3). There were, however, some small differences. The age band with highest odds of polypharmacy was 65 to 74 year olds (OR 9.24, 95% CI 8.99 to 9.50), with over 75s having slightly lower odds (OR 6.58, 95% CI 6.38 to 6.79). Also, the adjusted odds of being on polypharmacy followed a statistically significant trend downwards from most deprived to least deprived (OR 0.68, 95% CI 0.65 to 0.71). Adjusted effects for frailty, ethnicity and gender were not directly comparable to the unadjusted results, due to interaction terms. For instance, among White women, every additional eFI component was associated with 53% greater odds of being on polypharmacy (OR 1.53, 95% CI 1.53 to 1.54).

**Table 3:** Regression results. Mixed-effects multivariable logistic regression model. Outcome: polypharmacy, defined as being on ≥5 dispositions.

Characteristic	OR^*1*^	95% CI^*1*^	p-value
**Age group**			
18–44	—	—	
45–64	5.57	5.44, 5.71	<0.001
65–74	9.24	8.99, 9.50	<0.001
75+	6.58	6.38, 6.79	<0.001
**Ethnic Category**			
White	—	—	
Asian	0.98	0.96, 1.00	0.12
Black	0.82	0.79, 0.85	<0.001
Mixed	0.94	0.89, 1.00	0.064
Other	0.97	0.94, 1.01	0.11
Missing	0.17	0.15, 0.21	<0.001
**Gender**			
Female	—	—	
Male	1.26	1.24, 1.28	<0.001
**IMD Quintile**			
Most Deprived 1	—	—	
2	0.90	0.88, 0.93	<0.001
3	0.82	0.80, 0.84	<0.001
4	0.73	0.71, 0.76	<0.001
Least Deprived 5	0.68	0.65, 0.71	<0.001
**Number of eFI components (OR per additional component, 35 in total)**	1.53	1.53, 1.54	<0.001
Ethnic Category * no. eFI components			
Asian * eFI	1.05	1.05, 1.06	<0.001
Black * eFI	1.02	1.01, 1.03	<0.001
Mixed * eFI	1.04	1.02, 1.06	<0.001
Other * eFI	1.06	1.05, 1.07	<0.001
Missing * eFI	1.51	1.41, 1.61	<0.001
Gender * no. eFI components			
Male * eFI	1.10	1.09, 1.10	<0.001

^*1*^ OR = Odds Ratio, CI = Confidence Interval, * = interaction terms

At the average frailty level men had higher odds of polypharmacy than women (OR 1.26 95% CI 1.24 to 1.28). The interaction between gender and frailty showed that for every additional eFI component, men had a further 10% greater odds of polypharmacy (OR 1.10, 95% CI: 1.09, 1.10). The contrast between these results and the unadjusted finding that more women are on polypharmacy than men is due to differing distributions of frailty among men and women, with the mean number of eFI components being 1.72 for men and 2.68 for women.

At average eFI, those of Black ethnicity were significantly less likely to be on polypharmacy than those of White ethnicity (OR 0.82 95% CI 0.79 to 0.85). Odds of being on polypharmacy for those of Asian, Mixed and Other ethnicity were similar to those of White ethnicity. The effect of increasing frailty was greater for those of Black ethnicity, with an additional 1.02 (95% CI 1.01 to 1.03) increase in odds of polypharmacy for every additional eFI component, over and above the 53% increase per component for White women. This effect was seen for all non-White ethnicities.

**Fig 1:**
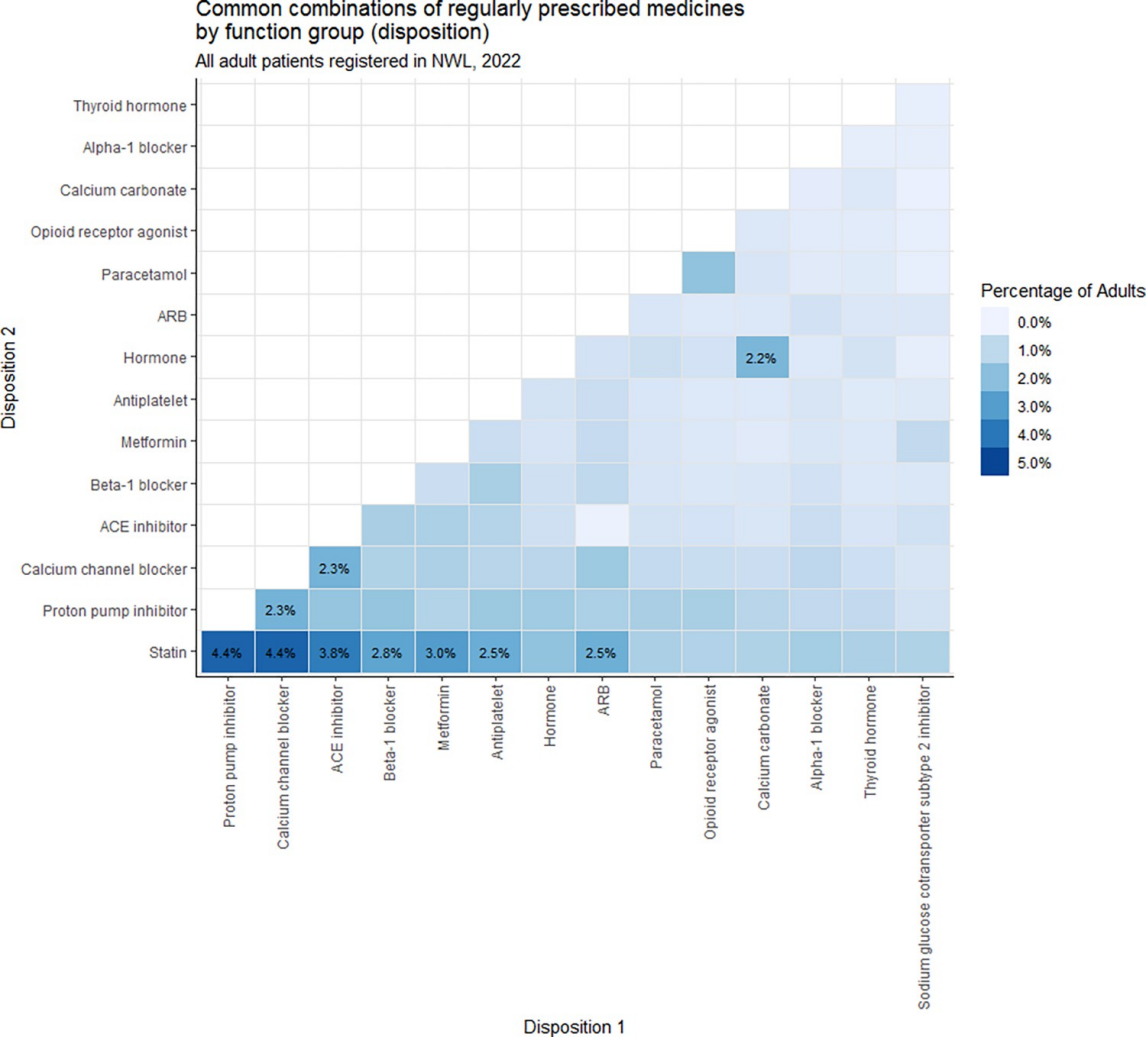
Disposition pair-wise combinations. Frequency distribution of the most commonly occurring pair-wise combinations of dispositions prescribed regularly during the study period. For example, 4.4% of the study population were regularly prescribed both a statin and a proton pump inhibitor during the study period.

The pair of dispositions most commonly prescribed regularly (4.4% of the study population) was statins (most commonly atorvastatin) with calcium channel blockers (most commonly amlodipine) (Fig 1). This was followed (4.4% of the study population) by statins with proton-pump inhibitors (most commonly omeprazole). Indeed, all of the top five most commonly prescribed pairings involved cardiovascular medications, including angiotensin-converting enzyme (ACE) inhibitors and beta blockers. Within dispositions commonly prescribed alongside others, there were also examples of pairings with relatively low frequency. For example, angiotensin II receptor blockers (ARBs) were co-prescribed with ACE inhibitors in 1,422 individuals (0.08% of the study population), a combination that is known to raise the risk of adverse drug reactions [28].

Costs associated with regular medications for all patients prescribed at least one regular medication over the study period totalled £78.03M (mean £150.83 per patient) (Table 4). The annual direct cost per patient for those on polypharmacy was 8.2 times greater than those not on polypharmacy (£370.89 and £45.31), despite the number of regular medications prescribed in these groups only being 3.6 times greater (8.7 and 2.4).

**Table 4:** Drug cost population summary. Total and mean direct annual drug costs of study population with 1 or more dispositions by the number of regular dispositions they were on within the study period; 1 to 4 dispositions, 5 or more dispositions, and the total within each demographic trait.

	Total cost for patients with 1–4 Regular Medications (Mean annual cost per patient)	Total cost for patients with > = 5 Regular Medications (Mean annual cost per patient)	Total cost for study population with >1 Regular Medication (Mean annual cost per patient)
**All Patients**	£15.84M (£45.31)	£62.19M (£370.89)	£78.03M (£150.83)
**Age Group**			
**18–44**	£4.60M (£42.68)	£3.05M (£333.58)	£7.65M (£65.42)
**45–64**	£6.60M (£43.94)	£18.61M (£332.27)	£25.21M (£22.23)
**65–74**	£2.50M (£44.74)	£15.61M (£344.73)	£18.11M (£178.99)
**75+**	£2.14M (£59.90)	£24.92M (£435.33)	£27.06M (£290.98)
**Ethnicity**			
**White**	£8.28M (£48.07)	£28.57M (£376.41)	£36.85M (£148.47)
**Asian**	£4.16M (£40.85)	£21.01M (£359.77)	£25.17M (£157.17)
**Black**	£1.48M (£46.00)	£6.36M (£396.34)	£7.84M (£162.49)
**Mixed**	£475.63K (£45.02)	£1.60M (£379.35)	£2.07M (£140.37)
**Other**	£1.37M (£44.53)	£4.61M (£356.79)	£5.98M (£136.92)
**Missing**	£77.38K (£36.80)	£37.43K (£204.56)	£114.82K (£50.23)
**Gender**			
**Female**	£8.26M (£40.80)	£31.65M (£360.30)	£39.91M (£137.48)
**Male**	£7.58M (£51.51)	£30.53M (£382.55)	£38.12M (£167.90)
**IMD Quintile**			
**Most Deprived 1**	£2.15M (£49.72)	£10.58M (£444.63)	£12.73M (£190.04)
**2**	£5.07M (£47.15)	£22.04M (£405.74)	£27.11M (£167.50)
**3**	£4.43M (£43.90)	£17.04M (£351.98)	£21.47M (£143.81)
**4**	£2.94M (£44.23)	£8.95M (£317.80)	£11.89M (£125.63)
**Least Deprived 5**	£1.26M (£39.82)	£3.57M (£275.41)	£4.83M (£108.34)

## Discussion

### Summary

This study of 1.7 million adults found overall prevalence of polypharmacy is 9.4%. Older age, greater frailty, and living in an area of higher socio-economic deprivation were independently associated with polypharmacy. Having adjusted for frailty, male gender was associated with polypharmacy. With average frailty, Black people were less likely than other ethnicities to be on polypharmacy. However, the effect of increasing frailty was greater for all non-White ethnicities.

Our results on direct cost of regular medications implicated in polypharmacy are the first derived from electronic health records in the UK, and demonstrate the considerable resources involved. Notably, the cost per patient on polypharmacy is greater than expected simply based on the number of regular medications prescribed, suggesting that patients on polypharmacy may be prescribed more expensive medication on average than those on fewer regular medications.

We developed a new method for identifying polypharmacy in primary care data, using the SNOMED CT ontology. This method enabled us to identify groups of medications by function in the body, count regularly prescribed medications and identify common combinations of medications prescribed for the same patient. The most common combinations included cardiovascular medications, including statins with calcium channel blockers or proton pump inhibitors. Combinations associated with a high risk of adverse side effects, such as angiotensin II receptor blockers (ARBs) with angiotensin-converting enzyme (ACE) inhibitors, occur with low frequency, but were still prescribed for some patients.

### Strengths and limitations

The WSIC dataset provided high coverage of the population and full coverage of primary care prescriptions. This minimised selection bias compared with a sampling approach. The population of NWL is large, diverse, and broadly representative of the demographics of the wider UK population, strengthening the external validity of the study [23]. Ethnicity was well documented in the study population, enabling better understanding of potential health inequalities.

Our method for identifying polypharmacy ensured medications only differing in dosage, and medications with the same function, were not double counted (S1 Box). For example, switching a patient from atorvastatin to simvastatin part way through the study period would not add to their count of regular medications, since both have the same disposition. We deliberately chose to count drugs by their function rather than by body system, as in the BNF, for two reasons. First, primary care EHR databases do not systematically record the indication for each prescription. Therefore it is not possible to reliably map each prescription onto the problem or disease, and hence body system, it was prescribed for. Second, this approach of counting distinct drug functions in the body has the advantage of highlighting cases where a large number of fundamentally different drugs are prescribed for the same patient, better reflecting complex polypharmacy. The approach is therefore well suited to describing polypharmacy from the important perspective of potential for drug-drug interactions. In contrast, this method is not suitable for identifying issues of therapeutic duplication involving two or more drugs in the same disposition group. This limitation is shared by other approaches using groups of medications to count polypharmacy, for example BNF chapters. The method is widely applicable to any SNOMED CT coded prescription data. Our mixed-effects multiple regression model enabled a precise understanding of independent associations between each variable and polypharmacy, and this is the first comparable study to separate out interactions between demographics and frailty.

This study was limited by the data, which were routinely collected and therefore dependent on coding by primary care teams. We had data on prescription but not dispensing of medication. Therefore, whilst we were able to quantify and describe patterns in polypharmacy as intentionally prescribed by a clinician, we were not able to characterise to what extent prescriptions were dispensed by a pharmacist or collected or used by patients. Patterns of polypharmacy as ingested or otherwise used by patients may differ from prescription patterns such as those identified in this study, for example where there are differences in adherence by demographics [29]. The eFI is a well-established and validated measure of frailty [27]. A 2023 systematic review found that eFI was the best-performing validated electronic measure of frailty, although the review only included one study using eFI [30]. However well the eFI performs as a measure of frailty, like all such measures it will not capture all aspects of frailty relevant to polypharmacy. Since this study is observational and does not establish causal explanations for the patterns identified, this is not a major limitation, but it is important to note that some of the variation associated with other variables may be partly explained by unmeasured frailty.

The NHS underwent radical changes during the height of the COVID-19 pandemic, for example many more GP consultations were conducted remotely [31]. All major public health measures, such as lockdown restrictions, were lifted by summer 2021 in England. However, it is possible that some differences in prescribing during this time persisted into the study period. However, given that the study period commenced six months after the lifting of restrictions, it is likely that the findings of this study reflect post-lockdown prescribing practices in the NHS and hence are relevant in informing policy and practice.

### Comparison with existing literature

Two previous studies from Scotland found higher rates of polypharmacy (five or more medications) than were identified in this study, Payne et al. finding approximately 17% prevalence in 2006, and Guthrie et al. 21% prevalence counting distinct chemical substances, and 7% counting BNF chapters, by 2010 [5, 16]. These studies were set in different populations, and used different methods, with Payne et al. using a simple count of prescriptions, rather than classifying drugs into categories, and Guthrie et al. using 355 subgroups of an amended British National Formulary (BNF) classification, splitting BNF chapters containing multiple drugs considered distinct and often co-prescribed. Similar to Payne et al. [5], an NHS report on polypharmacy counted by distinct medications and presented that 15% of people in England in 2019 were on five or more medications [19]. A study using electronic health records from the Spanish National Health System, using the Anatomical Therapeutic Chemical active substance classification, found a prevalence of 8.9% by 2015, more similar to the prevalence identified in this study [18]. All studies used different methods to characterise regularity of prescription in defining polypharmacy, impacting prevalence figures.

Patterns in polypharmacy recorded in the literature were broadly consistent with our findings, with greater prevalence associated with increasing age and higher levels of socio-economic deprivation. Payne et al. found increasing prevalence of polypharmacy with higher numbers of clinical conditions, consistent with our findings in relation to frailty [5]. Our finding that unadjusted prevalence of polypharmacy was greater among women than men was reversed on adjusting for number of frailty components. This reflects higher average levels of frailty among women compared with men, coupled with the strong association of frailty with polypharmacy. Payne et al. similarly found no statistically significant difference between men and women when adjusting for number of clinical conditions, where women were more likely to be prescribed more medications in univariate analysis [5]. Our finding of increased odds of polypharmacy with increasing socio-economic deprivation is consistent with previous studies [5, 16]. This consistency with existing literature supports the generalisability of the trends and patterns in our findings for populations and health systems that are similar to the National Health Service in England (NHS), and particularly Northwest London. There will of course be variation in prescribing practices both within and between such systems. It is likely these findings will not generalise to populations and health systems that differ significantly from the NHS, for example where healthcare policy, population needs, and available medications differ.

### Implications for research and practice

Our study shows that one in ten adults are on polypharmacy, and that whilst in many cases this may be beneficial, for some patients potentially problematic drug combinations may be prescribed. This supports the use of interventions to reduce problematic polypharmacy, such as structured medication review (SMR), as recommended by NHS England [32]. Such interventions create space for shared decision making between patients and clinicians, with potential to improve drug adherence, improve health outcomes, and reduce waste [33]. There is limited evidence on roll out of SMR and its effectiveness at population level [34]. Future research should quantify uptake of SMRs in practice, and examine their effectiveness at scale.

Our results provide evidence not only of well-known patterns of polypharmacy such as increasing prevalence with age, frailty, and lower socio-economic deprivation, but also of new associations, particularly in relation to interactions between frailty, and gender and ethnicity. This is the first study of large electronic health record data to examine the relationship between ethnicity and polypharmacy. Our findings indicate that the burden of polypharmacy becomes greater with increasing frailty to a larger extent among people of Black, Asian and Other ethnicities than it does among those of White ethnicity.

This differential effect of frailty for different genders and ethnicities suggests these interactions are important in research and in informing practice. A UK-based study of patients with dementia found differences in prescribing patterns by ethnicity, and another found variation in prescription of anti-osteoporosis drugs associated with income deprivation [35, 36]. Both are examples of differences in prescribing associated with demographics among patient groups who have higher than average frailty, and it is possible that the interaction effects found in this study are aggregations of these more specific patterns within condition or drug-specific subpopulations. Future research should explore causes of these effects, since this may inform strategies to improve care for patients on polypharmacy, including where they may be affected by health inequalities. Such strategies should be informed by real time analytics driven by routinely collected electronic health record data, to help focus intervention towards those with greatest need.

In this study we did not restrict attention to problematic polypharmacy, but rather set out to quantify the overall prevalence of polypharmacy and to understand variation by demographics and frailty. Future research should extend this analysis to examine problematic polypharmacy specifically, and quantify costs associated with problematic polypharmacy accounting for patient harm resulting from adverse events. This should include a focus on commonly co-prescribed medications and their interactions, especially any potentially inappropriate combinations as prescribed in practice in the population. Furthermore, benefits of medication should be incorporated into health economic analyses to provide the full picture.

Our method for counting distinct medications prescribed to each patient using primary care records may be used both in future research on polypharmacy, and in practice by regional or national health systems to measure and analyse polypharmacy with a view to improving quality of care.

## Supporting information

S1 Box Generating patients and GP prescriptions datasets. (DOCX)

S1 Fig Exclusion criteria–flow diagram. (DOCX)

S1 Table Gender-age population summary. (DOCX)

S2 Table Electronic Frailty Index (eFI) component population summary. (DOCX)

S3 Table Disposition frequency. (DOCX)

S4 Table Disposition coding source. (DOCX)

S5 Table Disposition medicatons: Statins (full list). (DOCX)

S6 Table Exclusion criteria–prescription medication dose forms. (DOCX)

S7 Table Exclusion criteria–active ingredients with no disposition. (DOCX)
