# Supplementary material for: Polypharmacy in primary care: A population-based retrospective cohort study of electronic health records
Source: PLoS One. 2024 Sep 4;19(9):e0308624. doi: 10.1371/journal.pone.0308624 (PMC11373791; doi:10.1371/journal.pone.0308624)
Supplement: S1 Box — (DOCX) [file pone.0308624.s001.docx]

S1 Box: Generating patients and GP prescriptions datasets


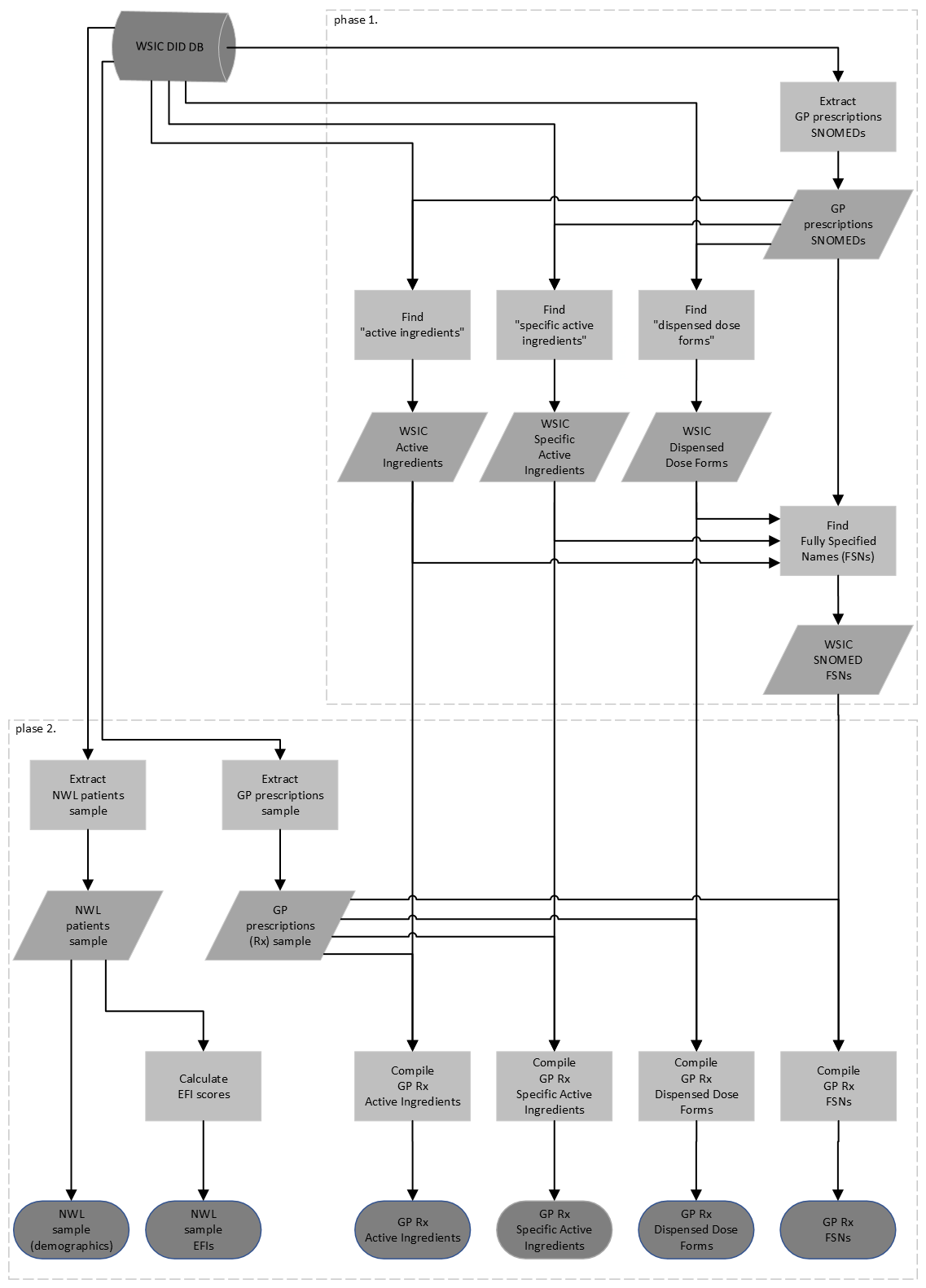


*NWL* – North West London; *WSIC DID DB* – Whole Systems Integrated Care De-IDentified Database; *SNOMED* – Systematized Nomenclature of Medicine; *FSN* – SNOMED Fully Specified Name; *EFI* – Electronic Frailty Index; *GP Rx* – GP Prescriptions.

The data used as input to the Polypharmacy project’s analysis were compiled in six separate datasets. These datasets were generated in several steps in two phases. In *Phase1* we processed available electronic GP prescription records to identify medicinal substances, ingredients, dose forms, and unique identifiable terms. In *Phase2*, we compiled a NWL patients sample with its demographic data and EFI scores, and a GP prescriptions sample with its relevant medications’ details.

Phase1.

We began the data compilation with the extraction of all unique pharmaceuticals found in GP prescription records available in the WSIC database. For each pharmaceutical, we then found its “active” or “specific active” ingredients. For those pharmaceuticals having no directly associated ingredients, we used their parent-child relationships inherent in the SNOMED hierarchy. The parent-child relationships were traversed up until an ingredient of a “parent” (a more common type of the pharmaceutical substance) was discovered. Along with the ingredients, we also extracted their “disposition”, the intended effect(s) a substance has on an organism.

Next, for each of the prescription pharmaceuticals, we identified their “dispensed dose form”, which itself was further classified as a “basic” or a “pharmaceutical” dose form. In the last step of this phase, we extracted the Fully Specified Names, the unique medical term, assigned to every SNOMED code collected in the preceding steps.

Phase 2.

As the first step in this phase, we extracted a sample of patients who were residents of one of the eight boroughs of North West London and were registered with a NWL GP practice. Each patient’s demographic data, such as age, ethnicity, gender, and IMD decile were also included. Next, for each patient in the sample, we calculated their Electronic Frailty Index score by using GP data recorded during our period of observation.

We then extracted a sample of electronic GP prescription records also collected during our observation period. Each prescribed medication was associated with the relevant ingredients, dispositions, dose forms and FSNs gathered in Phase1.

The two compiled patient datasets, one with demographics and one with EFI scores, along with the four prescription datasets, one for active ingredients, specific active ingredients, dose forms, and FSNs, formed the input data to the analysis phase of the project.
