# Supplementary material for: Polypharmacy in primary care: A population-based retrospective cohort study of electronic health records
Source: PLoS One. 2024 Sep 4;19(9):e0308624. doi: 10.1371/journal.pone.0308624 (PMC11373791; doi:10.1371/journal.pone.0308624)

S7 Figure: Exclusion Criteria – Flow Diagram

Flow diagram of effect of exclusion criteria on number of people represented within extracted data


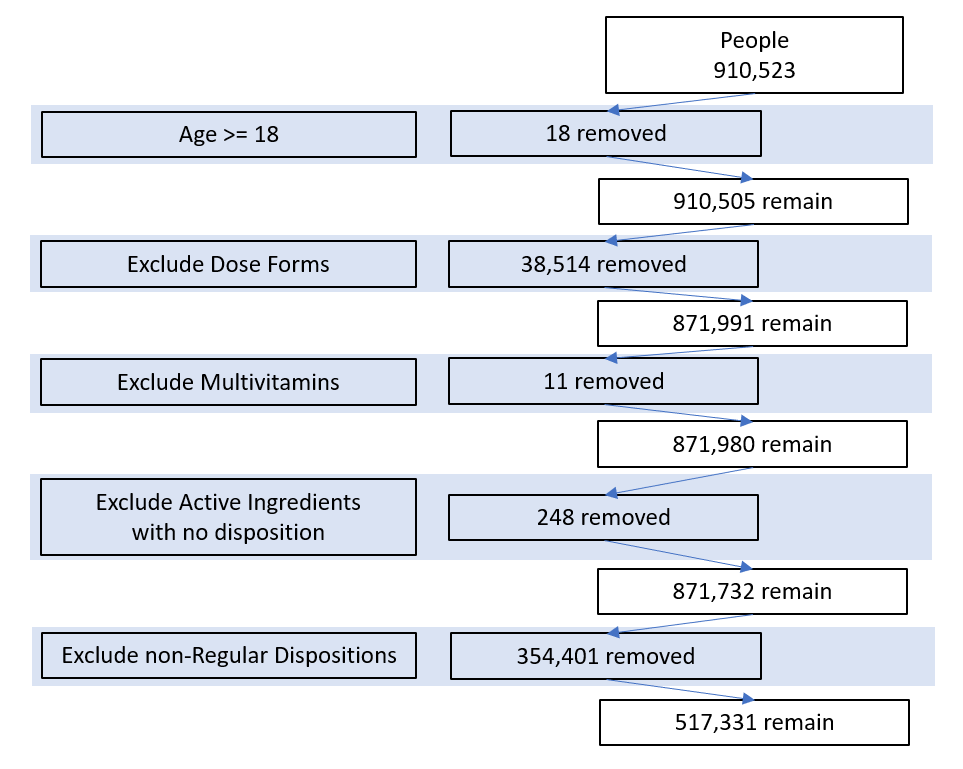

Supplement: S1 Fig — (DOCX) [file pone.0308624.s002.docx]
