# Supplementary material for: Polypharmacy in primary care: A population-based retrospective cohort study of electronic health records
Source: PLoS One. 2024 Sep 4;19(9):e0308624. doi: 10.1371/journal.pone.0308624 (PMC11373791; doi:10.1371/journal.pone.0308624)
Supplement: S1 Table — (DOCX) [file pone.0308624.s003.docx]

S2 Table: Gender-age population summary

Study population Gender-Age Group by the number of regular dispositions they were on within the study period, 0 dispositions, 1 to 4 dispositions, 5 or more dispositions, and total count of patients with that demographic trait.

| Gender  Age Group | Patients with 0 Regular Medications (%) | Patients with 1-4 Regular Medications (%) | Patients with >=5 Regular Medications (%) | Total Patient Count (%) |
| --- | --- | --- | --- | --- |
| Male | | | | |
| 18-44 | 481,210 (92.1%) | 37,772 (7.2%) | 3,768 (0.7%) | 522,750 |
| 45-64 | 192,513 (66.4%) | 68,949 (23.8%) | 28,480 (9.8%) | 289,942 |
| 65-74 | 21,384 (30.6%) | 25,655 (36.8%) | 22,750 (32.6%) | 69,789 |
| 75+ | 8,674 (17.9%) | 14,832 (30.7%) | 24,820 (51.4%) | 48,326 |
| Female | | | | |
| 18-44 | 389,636 (83.8%) | 69,989 (15.1%) | 5,373 (1.2%) | 464,998 |
| 45-64 | 141,839 (56.6%) | 81,304 (32.4%) | 27,533 (11.0%) | 250,676 |
| 65-74 | 21,817 (29.3%) | 30,237 (40.5%) | 22,519 (30.2%) | 74,573 |
| 75+ | 10,445 (16.4%) | 20,925 (32.8%) | 32,422 (50.8%) | 63,792 |
