# Supplementary material for: Polypharmacy in primary care: A population-based retrospective cohort study of electronic health records
Source: PLoS One. 2024 Sep 4;19(9):e0308624. doi: 10.1371/journal.pone.0308624 (PMC11373791; doi:10.1371/journal.pone.0308624)
Supplement: S2 Table — (DOCX) [file pone.0308624.s004.docx]

S3 Table: Electronic Frailty Index (eFI) component population summary

Study population count of patients with each eFI component by the number of regular dispositions they were on within the study period, 0 dispositions, 1 to 4 dispositions, 5 or more dispositions, and total count of patients with that demographic trait.

| EFI Component | Patients with 0 Regular Medications (%) | Patients with 1-4 Regular Medications (%) | Patients with >=5 Regular Medications (%) | Total Patient Count (%) |
| --- | --- | --- | --- | --- |
| Activity limitation | 2,529 (28.9%) | 2,191 (25.0%) | 4,036 (46.1%) | 8,756 |
| Anaemia & haematinic deficiency | 202,110 (46.2%) | 128,903 (29.5%) | 106,103 (24.3%) | 437,116 |
| Arthritis | 30,163 (22.8%) | 45,888 (34.7%) | 56,333 (42.6%) | 132,384 |
| Atrial fibrillation | 3,890 (11.2%) | 8,831 (25.4%) | 22,003 (63.4%) | 34,724 |
| Cerebrovascular disease | 5,225 (15.2%) | 9,618 (28.0%) | 19,526 (56.8%) | 34,369 |
| Chronic kidney disease | 10,044 (12.8%) | 22,836 (29.1%) | 45,715 (58.2%) | 78,595 |
| Diabetes | 27,093 (16.6%) | 57,490 (35.2%) | 78,615 (48.2%) | 163,198 |
| Dizziness | 55,078 (38.1%) | 48,316 (33.4%) | 41,333 (28.6%) | 144,727 |
| Dyspnoea | 38,874 (34.6%) | 33,305 (29.6%) | 40,240 (35.8%) | 112,419 |
| Falls | 24,437 (25.5%) | 26,386 (27.6%) | 44,862 (46.9%) | 95,685 |
| Foot problems | 16,989 (31.2%) | 16,392 (30.1%) | 21,089 (38.7%) | 54,470 |
| Fragility fracture | 21,110 (43.0%) | 13,363 (27.2%) | 14,640 (29.8%) | 49,113 |
| Hearing impairment | 40,211 (34.4%) | 36,627 (31.3%) | 40,145 (34.3%) | 116,983 |
| Heart failure | 3,176 (7.5%) | 7,053 (16.6%) | 32,279 (75.9%) | 42,508 |
| Heart valve disease | 1,771 (18.2%) | 2,532 (26.1%) | 5,410 (55.7%) | 9,713 |
| Housebound | 23,142 (36.7%) | 15,345 (24.3%) | 24,575 (39.0%) | 63,062 |
| Hypertension | 46,370 (16.6%) | 116,265 (41.6%) | 116,592 (41.8%) | 279,227 |
| Hypotension / syncope | 34,092 (43.4%) | 22,781 (29.0%) | 21,692 (27.6%) | 78,565 |
| Ischaemic heart disease | 67,716 (35.9%) | 54,450 (28.9%) | 66,426 (35.2%) | 188,592 |
| Memory & cognitive problems | 5,883 (14.8%) | 10,811 (27.2%) | 23,017 (58.0%) | 39,711 |
| Mobility and transfer problems | 6,191 (13.5%) | 10,190 (22.2%) | 29,522 (64.3%) | 45,903 |
| Osteoporosis | 10,299 (18.1%) | 20,167 (35.4%) | 26,496 (46.5%) | 56,962 |
| Parkinsonism & tremor | 783 (15.3%) | 1,444 (28.3%) | 2,880 (56.4%) | 5,107 |
| Peptic ulcer | 5,463 (28.4%) | 5,928 (30.8%) | 7,844 (40.8%) | 19,235 |
| Peripheral vascular disease | 1,222 (7.3%) | 3,435 (20.5%) | 12,133 (72.3%) | 16,790 |
| Requirement for care | 18,109 (33.1%) | 13,522 (24.7%) | 23,047 (42.2%) | 54,678 |
| Respiratory disease | 177,053 (49.9%) | 101,694 (28.6%) | 76,357 (21.5%) | 355,104 |
| Skin ulcer | 26,055 (39.6%) | 19,088 (29.0%) | 20,675 (31.4%) | 65,818 |
| Sleep disturbance | 35,749 (38.8%) | 30,300 (32.8%) | 26,189 (28.4%) | 92,238 |
| Social vulnerability | 100,603 (46.4%) | 57,734 (26.7%) | 58,247 (26.9%) | 216,584 |
| Thyroid disease | 68,378 (34.3%) | 78,425 (39.3%) | 52,688 (26.4%) | 199,491 |
| Urinary incontinence | 13,546 (23.2%) | 17,753 (30.5%) | 26,978 (46.3%) | 58,277 |
| Urinary system disease | 144,239 (45.7%) | 98,090 (31.1%) | 73,092 (23.2%) | 315,421 |
| Visual impairment | 51,608 (30.3%) | 53,963 (31.7%) | 64,847 (38.1%) | 170,418 |
| Weight loss & anorexia | 22,086 (37.8%) | 17,354 (29.7%) | 18,933 (32.4%) | 58,373 |
