# Supplementary material for: Polypharmacy in primary care: A population-based retrospective cohort study of electronic health records
Source: PLoS One. 2024 Sep 4;19(9):e0308624. doi: 10.1371/journal.pone.0308624 (PMC11373791; doi:10.1371/journal.pone.0308624)
Supplement: S3 Table — (DOCX) [file pone.0308624.s005.docx]

S4 Table: Disposition frequency

Dispositions prescribed within study period and number of patients regularly prescribed each disposition (descending frequency). Dispositions prescribed to less than 1000 patients are not listed.

| Disposition | Number of patients prescribed disposition |
| --- | --- |
| 3-hydroxy-3-methylglutaryl-coenzyme A reductase inhibitor | 217,380 |
| Hydrogen/potassium adenosine triphosphatase enzyme system inhibitor | 137,388 |
| Calcium channel blocker | 129,170 |
| Angiotensin-converting enzyme inhibitor | 99,149 |
| Serotonin reuptake inhibitor | 74,390 |
| Metformin | 68,151 |
| Angiotensin II receptor antagonist | 66,425 |
| Beta-1 adrenergic receptor antagonist | 65,640 |
| Thyroid hormone | 63,696 |
| Hormone | 60,025 |
| Antibacterial | 56,535 |
| Platelet aggregation inhibitor | 51,422 |
| Opioid receptor agonist | 47,219 |
| Paracetamol | 46,700 |
| Calcium carbonate | 40,873 |
| Alpha-1 adrenergic receptor antagonist | 39,918 |
| Adrenal cortex hormone | 38,796 |
| Laxative | 33,357 |
| Sodium glucose cotransporter subtype 2 inhibitor | 26,814 |
| Muscarinic receptor antagonist | 26,686 |
| Coagulation factor Xa inhibitor | 26,172 |
| Progesterone receptor agonist | 23,150 |
| Beta-2 adrenergic receptor agonist | 22,096 |
| Tricyclic antidepressant | 21,858 |
| P2Y12 G-protein-coupled platelet receptor antagonist | 21,557 |
| Dipeptidyl peptidase IV inhibitor | 21,004 |
| Histamine H1 receptor antagonist | 19,451 |
| Loop diuretic | 19,177 |
| Iron | 18,619 |
| Estrogen receptor agonist | 17,102 |
| Alpha adrenergic receptor antagonist | 17,076 |
| Gliclazide | 17,073 |
| Thiazide-like | 15,521 |
| Histamine receptor antagonist | 15,328 |
| Beta adrenergic receptor antagonist | 14,475 |
| Thiazide | 14,018 |
| Bisphosphonate | 13,622 |
| Immunomodulator | 13,232 |
| Folic acid | 12,878 |
| 5-alpha reductase inhibitor | 10,466 |
| Nitrates | 10,359 |
| Sodium channel blocker | 8,523 |
| Ezetimibe | 8,416 |
| Aldosterone receptor antagonist | 8,322 |
| Antimalarial | 7,698 |
| Xanthine oxidase inhibitor | 7,157 |
| Phosphodiesterase 5 inhibitor | 7,122 |
| Pregabalin | 7,100 |
| Leukotriene receptor antagonist | 6,983 |
| Antifungal | 6,835 |
| Sodium bicarbonate | 6,628 |
| Lubricating eye drops | 5,848 |
| Thiamine | 5,455 |
| Quetiapine | 5,145 |
| Beta-3 adrenergic receptor agonist | 5,087 |
| Olanzapine | 5,047 |
| Benzodiazepine | 4,796 |
| Coumarin | 4,767 |
| Potassium bicarbonate | 4,748 |
| Potassium | 4,615 |
| Mesalazine | 4,365 |
| Carbocisteine | 4,152 |
| Aluminium hydroxide | 4,139 |
| Carbonic anhydrase inhibitor | 4,122 |
| Aromatase inhibitor | 3,849 |
| Aripiprazole | 3,643 |
| Dopamine receptor agonist | 3,633 |
| Serotonin 5-hydroxytryptamine-1 receptor agonist | 3,633 |
| Vitamin B and/or vitamin B derivative | 3,461 |
| Zopiclone | 3,458 |
| Cyanocobalamin | 3,373 |
| Digoxin | 3,281 |
| Risperidone | 3,079 |
| Histamine H2 receptor antagonist | 2,931 |
| Alfacalcidol | 2,859 |
| Centrally acting acetylcholinesterase inhibitor | 2,796 |
| Levetiracetam | 2,723 |
| Betahistine | 2,585 |
| Carbamazepine | 2,567 |
| Mebeverine | 2,561 |
| Antiviral | 2,559 |
| Loperamide | 2,494 |
| Prochlorperazine | 2,313 |
| Adrenal cortex hormone | 2,309 |
| Melatonin | 2,115 |
| Gamma-aminobutyric acid A receptor agonist | 2,049 |
| Estrogen receptor antagonist | 1,948 |
| Mast cell stabilizer | 1,943 |
| Neprilysin inhibitor | 1,905 |
| Antimetabolite | 1,791 |
| N-methyl-D-aspartate receptor antagonist | 1,725 |
| Purine antagonist | 1,716 |
| Sodium chloride | 1,677 |
| Peroxisome proliferator-activated alpha receptor agonist | 1,466 |
| Pancreatic enzyme | 1,454 |
| Salicylic acid | 1,444 |
| Potassium channel activator | 1,426 |
| Ascorbic acid | 1,341 |
| Smoothened receptor agonist | 1,296 |
| Lipase inhibitor | 1,251 |
| Riboflavin | 1,243 |
| Vitamin D and/or vitamin D derivative | 1,234 |
| Lisdexamfetamine | 1,229 |
| Dopamine receptor antagonist | 1,182 |
| Glimepiride | 1,180 |
| Nicotinamide | 1,170 |
| Thiamine hydrochloride | 1,170 |
| Carbimazole | 1,133 |
| Amiodarone | 1,124 |
| Gonad regulating hormone | 1,084 |
| Tranexamic acid | 1,056 |
