# Supplementary material for: Polypharmacy in primary care: A population-based retrospective cohort study of electronic health records
Source: PLoS One. 2024 Sep 4;19(9):e0308624. doi: 10.1371/journal.pone.0308624 (PMC11373791; doi:10.1371/journal.pone.0308624)
Supplement: S4 Table — (DOCX) [file pone.0308624.s006.docx]

## S5 Table: Disposition coding source

Dispositions listed alphabetically and how they were classified. For medications with at least one active ingredient with a ‘SNOMED-CT’ code, this has been used. Medications with at least one active ingredient but no associated disposition within SNOMED-CT, these were classified either by ‘Manual’ groupings or by their ‘Ingredient’ name.

| Disposition | How Disposition Was Grouped |
| --- | --- |
| 3-hydroxy-3-methylglutaryl-coenzyme A reductase inhibitor | Actual |
| 4-aminobutyrate - 2-oxoglutarate transaminase inhibitor | Actual |
| 5-alpha reductase inhibitor | Actual |
| 5-hydroxytryptamine-3-receptor antagonist | Actual |
| A4beta2 acetylcholine receptor partial agonist | Actual |
| Acamprosate | Ingredient |
| Aceclofenac | Ingredient |
| Acetylcholine receptor antagonist | Actual |
| Acetylcysteine | Ingredient |
| Acipimox | Ingredient |
| Acitretin | Ingredient |
| Activated charcoal | Ingredient |
| Acute phase reactant | Actual |
| Adrenal cortex hormone | Manual |
| Adrenal cortex hormone | Actual |
| Aldehyde dehydrogenase inhibitor | Actual |
| Aldosterone receptor antagonist | Actual |
| Alfacalcidol | Ingredient |
| Allantoin | Ingredient |
| Alpha adrenergic receptor agonist | Actual |
| Alpha adrenergic receptor antagonist | Actual |
| Alpha tocopherol acetate | Ingredient |
| Alpha-1 adrenergic receptor antagonist | Actual |
| Alpha-2 adrenergic receptor agonist | Actual |
| Alpha-2 adrenergic receptor antagonist | Actual |
| Alpha-adrenergic and beta-adrenergic agonist | Actual |
| Alpha-amino-3-hydroxy-5-methyl-4-isoxazolepropionic acid receptor antagonist | Actual |
| Alpha-glucosidase inhibitor | Actual |
| Alprostadil | Ingredient |
| Aluminium chloride | Ingredient |
| Aluminium hydroxide | Ingredient |
| Aluminium oxide | Ingredient |
| Alverine | Ingredient |
| Alverine citrate | Ingredient |
| Aminophylline | Ingredient |
| Aminophylline hydrate | Ingredient |
| Amiodarone | Ingredient |
| Amisulpride | Ingredient |
| Ammonium salicylate | Ingredient |
| Amobarbital | Ingredient |
| Amobarbital sodium | Ingredient |
| Amylmetacresol | Ingredient |
| Androgen receptor agonist | Actual |
| Androgen receptor antagonist | Actual |
| Anethole | Ingredient |
| Angiotensin II receptor antagonist | Actual |
| Angiotensin-converting enzyme inhibitor | Actual |
| Anion exchange resin | Actual |
| Anthelmintic | Actual |
| Antibacterial | Actual |
| Antifungal | Actual |
| Antimalarial | Actual |
| Antimetabolite | Actual |
| Antimycobacterial | Actual |
| Antiprotozoal | Actual |
| Antiviral | Actual |
| Arachis oil | Ingredient |
| Arginine | Ingredient |
| Aripiprazole | Ingredient |
| Aromatase inhibitor | Actual |
| Ascorbic acid | Ingredient |
| Balsalazide | Ingredient |
| Balsam of Peru | Ingredient |
| Benzocaine | Ingredient |
| Benzodiazepine | Manual |
| Benzoyl peroxide | Ingredient |
| Benzydamine | Ingredient |
| Beta adrenergic receptor antagonist | Actual |
| Beta-1 adrenergic receptor antagonist | Actual |
| Beta-2 adrenergic receptor agonist | Actual |
| Beta-3 adrenergic receptor agonist | Actual |
| Betahistine | Ingredient |
| Bezafibrate | Ingredient |
| Biotin | Ingredient |
| Bismuth subsalicylate | Ingredient |
| Bisphosphonate | Manual |
| Bisphosphonate | Ingredient |
| Boric acid | Ingredient |
| Borneol | Ingredient |
| Brivaracetam | Ingredient |
| Bupropion | Ingredient |
| Bupropion hydrochloride | Ingredient |
| Buspirone | Ingredient |
| Calcium | Ingredient |
| Calcium acetate | Ingredient |
| Calcium carbonate | Ingredient |
| Calcium channel blocker | Actual |
| Calcium compound | Ingredient |
| Calcium electrolyte | Ingredient |
| Calcium gluconate | Ingredient |
| Calcium lactate | Ingredient |
| Calcium lactate gluconate | Ingredient |
| Calcium phosphate | Ingredient |
| Calcium polystyrene sulfonate | Ingredient |
| Calcium-sensing receptor activator | Actual |
| Camphene | Ingredient |
| Cannabinoid receptor agonist | Actual |
| Carbamazepine | Ingredient |
| Carbimazole | Ingredient |
| Carbocisteine | Ingredient |
| Carbohydrate | Ingredient |
| Carbon urea | Ingredient |
| Carbonic anhydrase inhibitor | Actual |
| Carboxylic acid and/or carboxylic acid derivative | Ingredient |
| Cariprazine | Ingredient |
| Catechol-O-methyltransferase inhibitor | Actual |
| Cation exchange resin | Actual |
| Cationic surfactant | Actual |
| Central alpha-2 adrenergic receptor agonist | Actual |
| Centrally acting acetylcholinesterase inhibitor | Actual |
| Centrally acting hypotensive agent | Ingredient |
| Chelating agent | Actual |
| Chloral hydrate | Ingredient |
| Chlordiazepoxide | Ingredient |
| Chlorhexidine | Ingredient |
| Chlorhexidine gluconate | Ingredient |
| Chlortalidone | Ingredient |
| Choline chloride | Ingredient |
| Cinchocaine | Ingredient |
| Cinchocaine hydrochloride | Ingredient |
| Cineole | Ingredient |
| Ciprofibrate | Ingredient |
| Citric acid | Ingredient |
| Clomethiazole | Ingredient |
| Clozapine | Ingredient |
| Coagulation factor Xa inhibitor | Actual |
| Coal tar | Ingredient |
| Coal tar extract | Ingredient |
| Coconut oil | Ingredient |
| Cod liver oil | Ingredient |
| Colchicine | Ingredient |
| Colesevelam | Ingredient |
| Copper | Ingredient |
| Coumarin | Manual |
| Cyanocobalamin | Ingredient |
| Cytochrome P450 2C19 inhibitor | Actual |
| Dantrolene | Ingredient |
| Dantron | Ingredient |
| Deflazacort | Ingredient |
| Dexamfetamine | Ingredient |
| Diazoxide | Ingredient |
| Dichlorobenzyl alcohol | Ingredient |
| Diclofenac | Ingredient |
| Diclofenac sodium | Ingredient |
| Digoxin | Ingredient |
| Dihydroxyphenylalanine decarboxylase inhibitor | Actual |
| Dipeptidyl peptidase IV inhibitor | Actual |
| Diphenoxylate | Ingredient |
| Direct thrombin inhibitor | Actual |
| Dopamine receptor agonist | Actual |
| Dopamine receptor antagonist | Actual |
| Dopamine receptor D2 antagonist | Actual |
| Dopamine reuptake inhibitor | Actual |
| Dosulepin | Ingredient |
| Dried aluminium hydroxide gel | Ingredient |
| Dronedarone | Ingredient |
| Electrolyte | Ingredient |
| Erdosteine | Ingredient |
| Ergocalciferol | Ingredient |
| Estrogen receptor agonist | Actual |
| Estrogen receptor antagonist | Actual |
| Ethosuximide | Ingredient |
| Ethyl chloride | Ingredient |
| Ethyl salicylate | Ingredient |
| Ezetimibe | Ingredient |
| Fenchone | Ingredient |
| Flecainide | Ingredient |
| Fludrocortisone | Ingredient |
| Fluocortolone caproate | Ingredient |
| Fluocortolone pivalate | Ingredient |
| Folic acid | Ingredient |
| Folinic acid | Ingredient |
| Gamma-aminobutyric acid A receptor agonist | Actual |
| Gamolenic acid | Ingredient |
| Gemfibrozil | Ingredient |
| Glacial acetic acid | Ingredient |
| Gliclazide | Ingredient |
| Glimepiride | Ingredient |
| Glipizide | Ingredient |
| Glucagon-like peptide 1 receptor agonist | Actual |
| Glucosamine | Ingredient |
| Glucose | Ingredient |
| Glutamate receptor antagonist | Actual |
| Glutaraldehyde | Ingredient |
| Glycerol | Ingredient |
| Glycol salicylate | Ingredient |
| Gonad regulating hormone | Actual |
| Gonadotropin releasing hormone receptor antagonist | Actual |
| Growth hormone receptor agonist | Actual |
| Guaifenesin | Ingredient |
| Guanylate cyclase 2C agonist | Actual |
| Haloperidol | Ingredient |
| Histamine H1 receptor antagonist | Actual |
| Histamine H2 receptor antagonist | Actual |
| Histamine receptor antagonist | Actual |
| Histone deacetylase inhibitor | Actual |
| Hormone | Actual |
| Hyaluronic acid | Ingredient |
| Hydralazine | Ingredient |
| Hydrogen/potassium adenosine triphosphatase enzyme system inhibitor | Actual |
| Hydrolase | Actual |
| Hydrotalcite | Ingredient |
| Hypothalamic inhibiting factor | Actual |
| Imidazoline receptor agonist | Actual |
| Immunomodulator | Actual |
| Integrase strand transfer inhibitor | Actual |
| Iron | Manual |
| Isometheptene | Ingredient |
| Isopropyl myristate | Ingredient |
| Isotretinoin | Ingredient |
| Ivabradine | Ingredient |
| Kaolin | Ingredient |
| Lacosamide | Ingredient |
| Lactic acid | Ingredient |
| Lanthanum carbonate | Ingredient |
| Laxative | Manual |
| Lecithin | Ingredient |
| Leukotriene receptor antagonist | Actual |
| Levetiracetam | Ingredient |
| Levocarnitine | Ingredient |
| Levomenthol | Ingredient |
| Levomepromazine | Ingredient |
| Lipase inhibitor | Actual |
| Lisdexamfetamine | Ingredient |
| Lithium carbonate | Ingredient |
| Lithium citrate | Ingredient |
| Lofepramine | Ingredient |
| Loop diuretic | Manual |
| Loperamide | Ingredient |
| Lubricating eye drops | Manual |
| Magnesium alginate | Ingredient |
| Magnesium aspartate | Ingredient |
| Magnesium carbonate | Ingredient |
| Magnesium citrate | Ingredient |
| Magnesium glycerophosphate | Ingredient |
| Magnesium hydroxide | Ingredient |
| Magnesium malate | Ingredient |
| Magnesium oxide | Ingredient |
| Magnesium sulfate | Ingredient |
| Manganese and/or manganese compound | Ingredient |
| Mast cell stabilizer | Actual |
| Mebeverine | Manual |
| Mefenamic acid | Ingredient |
| Melatonin | Ingredient |
| Melatonin agonist | Actual |
| Menadiol | Ingredient |
| Menthol | Ingredient |
| Mesalazine | Ingredient |
| Metformin | Manual |
| Methocarbamol | Ingredient |
| Methyl nicotinate | Ingredient |
| Methyl salicylate | Ingredient |
| Methylcellulose-450 | Ingredient |
| Methylphenidate | Ingredient |
| Methylphenidate hydrochloride | Ingredient |
| Methylprednisolone | Ingredient |
| Minoxidil | Ingredient |
| Misoprostol | Ingredient |
| Mitotic inhibitor | Actual |
| Mometasone furoate monohydrate | Ingredient |
| Monoamine oxidase A inhibitor | Actual |
| Monoamine oxidase B inhibitor | Actual |
| Monoamine oxidase inhibitor | Actual |
| Monobasic sodium phosphate | Ingredient |
| Muscarinic receptor agonist | Actual |
| Muscarinic receptor antagonist | Actual |
| Naftidrofuryl | Ingredient |
| Neprilysin inhibitor | Actual |
| Neurokinin 1 receptor antagonist | Actual |
| Nicotinamide | Ingredient |
| Nicotinic acid | Ingredient |
| Nicotinic receptor agonist | Actual |
| Nitrates | Manual |
| N-methyl-D-aspartate receptor antagonist | Actual |
| Nonionic surfactant | Actual |
| Norepinephrine reuptake inhibitor | Actual |
| Olanzapine | Ingredient |
| Olsalazine | Ingredient |
| Opioid receptor agonist | Actual |
| Opioid receptor antagonist | Actual |
| Opioid receptor partial agonist | Actual |
| Oxerutins | Ingredient |
| Oxetacaine | Ingredient |
| Oxidase | Actual |
| P2Y12 G-protein-coupled platelet receptor antagonist | Actual |
| Pancreatic enzyme | Actual |
| Pancreatic hormone | Actual |
| Pantothenate kinase inhibitor | Actual |
| Pantothenic acid | Ingredient |
| Paracetamol | Ingredient |
| Paraldehyde | Ingredient |
| Paricalcitol | Ingredient |
| Pentosan polysulfate | Ingredient |
| Pentoxifylline | Ingredient |
| Peppermint oil | Ingredient |
| Periciazine | Ingredient |
| Peroxisome proliferator-activated alpha receptor agonist | NA |
| Peroxisome proliferator-activated gamma receptor agonist | Actual |
| Phenobarbital | Ingredient |
| Phosphate | Ingredient |
| Phosphodiesterase 4 inhibitor | Actual |
| Phosphodiesterase 5 inhibitor | Actual |
| Phosphodiesterase inhibitor | Actual |
| Phytomenadione | Ingredient |
| Pinene | Ingredient |
| Piracetam | Ingredient |
| Plant fiber | Ingredient |
| Platelet aggregation inhibitor | Actual |
| Potassium | Ingredient |
| Potassium aminobenzoate | Ingredient |
| Potassium bicarbonate | Ingredient |
| Potassium channel activator | Actual |
| Potassium chloride | Ingredient |
| Potassium citrate | Ingredient |
| Potassium exchange resin | Actual |
| Potassium permanganate | Ingredient |
| Povidone iodine | Ingredient |
| Pregabalin | Ingredient |
| Primidone | Ingredient |
| Prochlorperazine | Ingredient |
| Progesterone receptor agonist | Actual |
| Promazine | Ingredient |
| Propafenone | Ingredient |
| Propylthiouracil | Ingredient |
| Prostaglandin-endoperoxide synthase inhibitor | Actual |
| Prostaglandin-endoperoxide synthase isoform 2 inhibitor | Actual |
| Protein-tyrosine kinase inhibitor | Actual |
| Psyllium | Ingredient |
| Purine antagonist | Actual |
| Pyridoxine | Ingredient |
| Quaternary ammonium compound | Ingredient |
| Quetiapine | Ingredient |
| Reboxetine | Ingredient |
| Renal hormone | Actual |
| Renin inhibitor | Actual |
| Repaglinide | Ingredient |
| Retinol | Ingredient |
| Reverse transcriptase inhibitor | Actual |
| Reversible anticholinesterase | Actual |
| Riboflavin | Ingredient |
| Ribonucleoside-diphosphate reductase inhibitor | Actual |
| Risperidone | Ingredient |
| Rufinamide | Ingredient |
| Salicylic acid | Ingredient |
| Scabicide | Actual |
| Secobarbital | Ingredient |
| Secobarbital sodium | Ingredient |
| Selective estrogen receptor modulator | Actual |
| Selective progesterone receptor modulator | Actual |
| Selenium | Ingredient |
| Serotonin 5-hydroxytryptamine-1 receptor agonist | Actual |
| Serotonin reuptake inhibitor | Actual |
| Sesame oil | Ingredient |
| Smoothened receptor agonist | Actual |
| Sodium bicarbonate | Ingredient |
| Sodium carbonate | Ingredient |
| Sodium channel blocker | Actual |
| Sodium chloride | Ingredient |
| Sodium feredetate | Ingredient |
| Sodium glucose cotransporter subtype 2 inhibitor | Actual |
| Sodium hyaluronate | Ingredient |
| Somatropin(rbe) | Ingredient |
| Stiripentol | Ingredient |
| Strontium ranelate | Ingredient |
| Sucralfate | Ingredient |
| Surfactant | Actual |
| Thiamine | Ingredient |
| Thiamine hydrochloride | Ingredient |
| Thiazide | Manual |
| Thiazide-like | Manual |
| Thrombopoietin receptor agonist | Actual |
| Thyroid hormone | Actual |
| Tizanidine | Ingredient |
| Tolbutamide | Ingredient |
| Torasemide | Ingredient |
| Toxin | Actual |
| Tranexamic acid | Ingredient |
| Trazodone | Ingredient |
| Tretinoin | Ingredient |
| Triamcinolone | Ingredient |
| Triamcinolone acetonide | Ingredient |
| Triclosan | Ingredient |
| Tricyclic antidepressant | Manual |
| Trifluoperazine | Ingredient |
| Tryptophan | Ingredient |
| Tumor necrosis factor alpha inhibitor | Actual |
| Ubidecarenone | Ingredient |
| Undecenoate | Ingredient |
| Urea | Ingredient |
| Ursodeoxycholic acid | Ingredient |
| Valerian root extract | Ingredient |
| Valine | Ingredient |
| Vasopressin receptor agonist | Actual |
| Vesicular monoamine transporter 2 inhibitor | Actual |
| Vitamin B and/or vitamin B derivative | Ingredient |
| Vitamin D and/or vitamin D derivative | Ingredient |
| Vitamin E and/or vitamin E derivative | Ingredient |
| Xanthine oxidase inhibitor | Actual |
| Xipamide | Ingredient |
| Zinc acetate | Ingredient |
| Zinc citrate | Ingredient |
| Zinc oxide | Ingredient |
| Zinc sulfate | Ingredient |
| Zinc undecylenate | Ingredient |
| Zonisamide | Ingredient |
| Zopiclone | Ingredient |
