# Supplementary material for: Polypharmacy in primary care: A population-based retrospective cohort study of electronic health records
Source: PLoS One. 2024 Sep 4;19(9):e0308624. doi: 10.1371/journal.pone.0308624 (PMC11373791; doi:10.1371/journal.pone.0308624)
Supplement: S5 Table — (DOCX) [file pone.0308624.s007.docx]

S6 Table: Disposition Medications: Statins (Full List)

| Medication  (Defined as ‘Product’ in SNOMED CT) | Ingredient | Patient Count |
| --- | --- | --- |
| Atorvastatin 20mg tablets | Atorvastatin | 92,237 |
| Atorvastatin 40mg tablets | Atorvastatin | 52,988 |
| Atorvastatin 10mg tablets | Atorvastatin | 19,845 |
| Atorvastatin 80mg tablets | Atorvastatin | 16,644 |
| Product containing precisely simvastatin 20 milligram/1 each conventional release oral tablet | Simvastatin | 14,955 |
| Product containing precisely simvastatin 40 milligram/1 each conventional release oral tablet | Simvastatin | 13,640 |
| Product containing precisely rosuvastatin (as rosuvastatin calcium) 5 milligram/1 each conventional release oral tablet | Rosuvastatin | 5,367 |
| Product containing precisely rosuvastatin (as rosuvastatin calcium) 10 milligram/1 each conventional release oral tablet | Rosuvastatin | 5,121 |
| Product containing precisely rosuvastatin (as rosuvastatin calcium) 20 milligram/1 each conventional release oral tablet | Rosuvastatin | 3,968 |
| Product containing precisely simvastatin 10 milligram/1 each conventional release oral tablet | Simvastatin | 2,748 |
| Product containing precisely pravastatin sodium 20 milligram/1 each conventional release oral tablet | Pravastatin | 2,015 |
| Product containing precisely pravastatin sodium 40 milligram/1 each conventional release oral tablet | Pravastatin | 1,705 |
| Product containing precisely pravastatin sodium 10 milligram/1 each conventional release oral tablet | Pravastatin | 1,395 |
| Product containing precisely rosuvastatin (as rosuvastatin calcium) 40 milligram/1 each conventional release oral tablet | Rosuvastatin | 1,036 |
| Lipitor 20mg tablets (Viatris UK Healthcare Ltd) | Atorvastatin | 176 |
| Atorvastatin 20mg tablets (Teva UK Ltd) | Atorvastatin | 147 |
| Product containing precisely simvastatin 80 milligram/1 each conventional release oral tablet | Simvastatin | 135 |
| Lipitor 10mg tablets (Viatris UK Healthcare Ltd) | Atorvastatin | 124 |
| Rosuvastatin 5mg capsules | Rosuvastatin | 105 |
| Lipitor 40mg tablets (Viatris UK Healthcare Ltd) | Atorvastatin | 100 |
| Rosuvastatin 10mg capsules | Rosuvastatin | 98 |
| Fluvastatin 20mg capsules | Fluvastatin | 97 |
| Crestor 5mg tablets (AstraZeneca) | Rosuvastatin | 85 |
| Rosuvastatin 20mg capsules | Rosuvastatin | 84 |
| Fluvastatin 40mg capsules | Fluvastatin | 71 |
| Crestor 10mg tablets (AstraZeneca) | Rosuvastatin | 71 |
| Atorvastatin 60mg tablets | Atorvastatin | 51 |
| Atorvastatin 40mg tablets (Teva UK Ltd) | Atorvastatin | 43 |
| Atorvastatin 20mg chewable tablets sugar free | Atorvastatin | 37 |
| Crestor 20mg tablets (AstraZeneca) | Rosuvastatin | 37 |
| Lipitor 80mg tablets (Viatris UK Healthcare Ltd) | Atorvastatin | 36 |
| Atorvastatin 10mg tablets (Teva UK Ltd) | Atorvastatin | 33 |
| Fluvastatin 80mg modified-release tablets | Fluvastatin | 17 |
| Rosuvastatin 40mg capsules | Rosuvastatin | 17 |
| Atorvastatin 30mg tablets | Atorvastatin | 16 |
| Atorvastatin 10mg chewable tablets sugar free | Atorvastatin | 15 |
| Atorvastatin 80mg tablets (Teva UK Ltd) | Atorvastatin | 14 |
| Product containing precisely ezetimibe 10 milligram and simvastatin 40 milligram/1 each conventional release oral tablet | Simvastatin | 14 |
| Crestor 40mg tablets (AstraZeneca) | Rosuvastatin | 10 |
| Atorvastatin 20mg tablets (Dexcel-Pharma Ltd) | Atorvastatin | 9 |
| Atorvastatin 20mg tablets (Wockhardt UK Ltd) | Atorvastatin | 9 |
| Zocor 20mg tablets (Organon Pharma (UK) Ltd) | Simvastatin | 9 |
| Atorvastatin 20mg/5ml oral suspension sugar free | Atorvastatin | 8 |
| Atorvastatin 20mg/5ml oral suspension | Atorvastatin | 7 |
| Zocor 10mg tablets (Organon Pharma (UK) Ltd) | Simvastatin | <=5 |
| Simvastatin 40mg/5ml oral suspension sugar free | Simvastatin | <=5 |
| Simvastatin 20mg/5ml oral suspension sugar free | Simvastatin | <=5 |
| Product containing precisely ezetimibe 10 milligram and simvastatin 20 milligram/1 each conventional release oral tablet | Simvastatin | <=5 |
| Zocor 40mg tablets (Organon Pharma (UK) Ltd) | Simvastatin | <=5 |
| Atorvastatin 20mg/5ml oral solution | Atorvastatin | <=5 |
| Lipitor 10mg chewable tablets (Viatris UK Healthcare Ltd) | Atorvastatin | <=5 |
| Lipitor 20mg chewable tablets (Viatris UK Healthcare Ltd) | Atorvastatin | <=5 |
| Atorvastatin 20mg tablets (Aspire Pharma Ltd) | Atorvastatin | <=5 |
| Rosuvastatin 15mg tablets | Rosuvastatin | <=5 |
| Atorvastatin 10mg/5ml oral suspension | Atorvastatin | <=5 |
| Atorvastatin 40mg tablets (Dexcel-Pharma Ltd) | Atorvastatin | <=5 |
| Atorvastatin 10mg tablets (Aspire Pharma Ltd) | Atorvastatin | <=5 |
| Atorvastatin 80mg tablets (Almus Pharmaceuticals Ltd) | Atorvastatin | <=5 |
| Rosuvastatin 5mg tablets (Teva UK Ltd) | Rosuvastatin | <=5 |
| Simvastatin 20mg tablets (Approved Prescription Services) | Simvastatin | <=5 |
| Simvastatin 40mg tablets (Sandoz Ltd) | Simvastatin | <=5 |
| Pravastatin 20mg tablets (Pliva Pharma Ltd) | Pravastatin | <=5 |
| Simvastatin 20mg tablets (Pliva Pharma Ltd) | Simvastatin | <=5 |
| Fluvastatin 40mg capsules (Zentiva Pharma UK Ltd) | Fluvastatin | <=5 |
| Simvastatin 20mg/5ml oral suspension sugar free (Rosemont Pharmaceuticals Ltd) | Simvastatin | <=5 |
| Atorvastatin 10mg tablets (Dexcel-Pharma Ltd) | Atorvastatin | <=5 |
| Atorvastatin 40mg tablets (Aspire Pharma Ltd) | Atorvastatin | <=5 |
| Atorvastatin 80mg tablets (Aspire Pharma Ltd) | Atorvastatin | <=5 |
| Atorvastatin 10mg tablets (Wockhardt UK Ltd) | Atorvastatin | <=5 |
| Atorvastatin 40mg tablets (Wockhardt UK Ltd) | Atorvastatin | <=5 |
| Atorvastatin 20mg tablets (Dr Reddy's Laboratories (UK) Ltd) | Atorvastatin | <=5 |
| Atorvastatin 20mg tablets (Almus Pharmaceuticals Ltd) | Atorvastatin | <=5 |
| Atorvastatin 40mg tablets (Almus Pharmaceuticals Ltd) | Atorvastatin | <=5 |
| Fenofibrate 145mg / Simvastatin 20mg tablets | Simvastatin | <=5 |
| Rosuvastatin 20mg tablets (Teva UK Ltd) | Rosuvastatin | <=5 |
| Rosuvastatin 10mg capsules (Sun Pharmaceutical Industries Europe B.V.) | Rosuvastatin | <=5 |
| Product containing precisely ezetimibe 10 milligram and simvastatin 80 milligram/1 each conventional release oral tablet | Simvastatin | <=5 |
| Simvastatin 40mg tablets (C P Pharmaceuticals Ltd) | Simvastatin | <=5 |
| Simvastatin 20mg tablets (Sandoz Ltd) | Simvastatin | <=5 |
| Pravastatin 40mg tablets (Ranbaxy (UK) Ltd) | Pravastatin | <=5 |
| Lescol 20mg capsules (Novartis Pharmaceuticals UK Ltd) | Fluvastatin | <=5 |
| Inegy 10mg/40mg tablets (Organon Pharma (UK) Ltd) | Simvastatin | <=5 |
