# Supplementary material for: Polypharmacy in primary care: A population-based retrospective cohort study of electronic health records
Source: PLoS One. 2024 Sep 4;19(9):e0308624. doi: 10.1371/journal.pone.0308624 (PMC11373791; doi:10.1371/journal.pone.0308624)
Supplement: S6 Table — (DOCX) [file pone.0308624.s008.docx]

S8 Table: Exclusion Criteria – Prescription Medication Dose Forms

List of Dose Forms excluded from the analysis and the number of prescription medications they were linked to.

| **Dose Form** | **Reason for exclusion** | **Frequency** |
| --- | --- | --- |
| Conventional release eye drops (dose form) | Eye drop | 430 |
| Cream (basic dose form) | Cream | 372 |
| Ointment (basic dose form) | Ointment | 186 |
| Gel (basic dose form) | Gel | 169 |
| Basic dose form with liquid state of matter (basic dose form) | Topical treatments | 140 |
| Impregnated dressing (qualifier value) | Dressings | 44 |
| Conventional release ear drops (dose form) | Ear drop | 36 |
| Conventional release oromucosal solution for mouthwash (dose form) | Mouthwashes | 32 |
| Bath additive (qualifier value) | Topical treatments | 31 |
| Shampoo (qualifier value) | Topical treatments | 31 |
| Irrigation solution (qualifier value) | Irrigation/topical use/external | 29 |
| Conventional release eye ointment (dose form) | Ointment | 27 |
| Pessary (basic dose form) | Locally acting | 27 |
| Conventional release oromucosal gel (dose form) | Gel | 22 |
| Conventional release nasal drops (dose form) | Nasal drops | 17 |
| Stick (basic dose form) | Local use | 17 |
| Medicated plaster (qualifier value) | Topical treatments | 16 |
| Paste (basic dose form) | Topical treatments | 15 |
| Conventional release eye gel (dose form) | Gel | 13 |
| Paint (qualifier value) | Topical treatments | 11 |
| Conventional release oral gel (dose form) | Gel | 10 |
| Medicated nail lacquer (qualifier value) | Topical treatments | 7 |
| Conventional release dental gel (dose form) | Gel | 7 |
| Conventional release intravesical solution (dose form) | Mostly bladder instillations | 6 |
| Ear/eye drops solution (qualifier value) | Eye/ear drop | 5 |
| Ear/eye/nose drops solution (qualifier value) | Eye/ear/nose drops | 5 |
| Conventional release vaginal gel (dose form) | Gel | 5 |
| Conventional release solution for cutaneous spray (dose form) | Topical treatments | 4 |
| Modified-release drops (qualifier value) | Eye drop | 3 |
| Powder and suspension for suspension for injection (qualifier value) | Vaccines/injectable | 3 |
| Irrigation (qualifier value) | Irrigation | 3 |
| Wash (qualifier value) | Topical treatments | 3 |
| Conventional release nasal ointment (dose form) | Ointment | 3 |
| Conventional release dental suspension (dose form) | Topical treatments | 2 |
| Conventional release cutaneous solution (dose form) | Topical treatments | 2 |
| Conventional release cutaneous emulsion (dose form) | Topical treatments | 2 |
| Conventional release rectal ointment (dose form) | Ointment | 2 |
| Gastroenteral liquid (qualifier value) |  | 1 |
| Conventional release gas for inhalation (dose form) | Inhaled gas | 1 |
| Pastille (basic dose form) | Topical treatments | 1 |
