# Supplementary material for: Polypharmacy in primary care: A population-based retrospective cohort study of electronic health records
Source: PLoS One. 2024 Sep 4;19(9):e0308624. doi: 10.1371/journal.pone.0308624 (PMC11373791; doi:10.1371/journal.pone.0308624)
Supplement: S7 Table — (DOCX) [file pone.0308624.s009.docx]

S9 Table: Exclusion Criteria – Active Ingredients with no Disposition

Active Ingredients with no disposition excluded from the analysis and the number of prescription medications they were linked to.

| **Ingredient** | **Frequency** |
| --- | --- |
| Mineral oil | 251148 |
| White paraffin | 153076 |
| Alginic acid | 57826 |
| Sodium alginate | 55666 |
| Antigen of Influenza virus | 45630 |
| Yellow paraffin | 33598 |
| Nitrogen and nitrogen compound | 30281 |
| White petrolatum | 21326 |
| Antigen of Salmonella enterica subspecies enterica serovar Typhi | 17093 |
| Paraffin | 16732 |
| Olive oil | 14549 |
| Antigen of Hepatitis A virus | 10114 |
| Antigen of Streptococcus pneumoniae | 5574 |
| Soya oil | 4418 |
| Antigen of Hepatitis B virus | 2078 |
| Antigen of Human alphaherpesvirus 3 | 1895 |
| Antigen of Neisseria meningitidis | 1440 |
| Water | 1425 |
| Silicone | 1264 |
| Antigen of Yellow fever virus | 923 |
| Antigen of Vibrio cholerae | 493 |
| Antigen of Rabies lyssavirus | 450 |
| Antigen of Human poliovirus | 319 |
| Antigen of Rotavirus | 176 |
| Antigen of Human papillomavirus | 137 |
| Antigen of Haemophilus influenzae type B | 112 |
| Antigen of Japanese encephalitis virus | 77 |
| Antigen of Tick-borne encephalitis virus | 34 |
| Substance categorized by structure | 32 |
| Cornflour | 11 |
| Paraffin wax | <6 |
